# Expression of MicroRNA-1281, C-Reactive Protein, and Renal Function in Individuals with Abdominal Aortic Aneurysm and their Clinical Correlation after Endovascular Repair

**DOI:** 10.21470/1678-9741-2020-0268

**Published:** 2021

**Authors:** Lais Missae, Breno Rossoni, Emanuel Junio Ramos Tenorio, Maurício Serra Ribeiro, Daniela Tirapelli, Edwaldo Edner Joviliano

**Affiliations:** 1 Division of Vascular and Endovascular Surgery, Department of Surgery and Anatomy, Clinical Hospital of Ribeirão Preto, University of São Paulo, Ribeirão Preto, São Paulo, Brazil.; 2 Department of Anatomy, Ribeirão Preto Medical School, University of São Paulo, Ribeirão Preto, São Paulo, Brazil.

**Keywords:** MicroRNAs, Aortic Aneurysm, Abdominal, Endovascular Procedures, C-Reactive Protein, Biomarkers

## Abstract

**Introduction:**

The treatment of infrarenal aortic aneurysms has changed in the last three decades. Endovascular aneurysm repair (EVAR) has become the primary treatment option in anatomically suitable patients with infrarenal aortic aneurysms. However, there is no serum biomarker to be used in EVAR follow-up.

**Methods:**

This is a prospective single-centre study of 30 consecutive patients with abdominal aortic aneurysm (AAA) who underwent EVAR. Serum dosages of micro ribonucleic acid 1281 (miRNA-1281), creatinine, total cholesterol, triglycerides, and C-reactive protein (CRP) were evaluated and angiotomographic evaluations were performed preoperatively and six months after the intervention.

**Results:**

There was a hyperexpression of miRNA-1281 in patients with AAA and a significant reduction of it after EVAR, from 1.66-fold before EVAR to 0.27 after the procedure (*P*<0.0001). MiRNA-1281 expression was not influenced by renal function (creatinine: 1.14±0.29, *P*=0.68), total cholesterol (179.9±59.9, *P*=0.22), or CRP (1.17±3.5; *P*=0.48). There is correlation between AAA size and CRP serum levels, however there was no statically significant reduction of CRP after EVAR.

**Discussion:**

MiRNA-1281 expression may be influenced by cholesterol, triglycerides levels, and renal function. We found no difference in these markers before and six months after EVAR. However, miRNA-1281 presents a significant reduction in patients with no follow-up complications. We hypothesize that miRNA-1281 expression may be related to aortic wall stress or flow changes.

**Conclusion:**

MiRNA-1281 may contribute as a possible marker of EVAR follow-up.

**Table:** 

Abbreviations, acronyms & symbols				
**AAA**	**= Abdominal aortic aneurysm**		**miRNA**	**= Micro ribonucleic acid**
**AMPK**	**= AMP-activated protein kinase**		**MMP**	**= Matrix metalloproteinase**
**AngII**	**= Angiotensin II**		**PAD**	**= Peripheral arterial disease**
**AP-2**	**= Activator protein 2**		**PCR**	**= Polymerase chain reaction**
**cDNA**	**= Complementary deoxyribonucleic acid**		**RQ-PCR**	**= Real-time quantitative polymerase chain reaction**
**CRP**	**= C-reactive protein**		**RNA**	**= Ribonucleic acid**
**CT**	**= Computed tomography**		**RT**	**= Reverse transcription**
**DEPC**	**= Diethyl pyrocarbonate**		**SAH**	**= Systemic arterial hypertension**
**EDTA**	**= Ethylenediaminetetraacetic acid**		**USA**	**= United States of America**
**EVAR**	**= Endovascular aneurysm repair**			

## INTRODUCTION

The prevalence of abdominal aortic aneurysm (AAA) depends on associated risk factors that include advanced age, male gender, white race, positive family history, smoking, hypertension, hypercholesterolaemia, peripheral vascular occlusive disease, and coronary artery disease. AAA progression is not linear, but usually presents points of acceleration which can appear at any time. Conversely, aortic dilatations can remain stable and asymptomatic for many years, during which aged patients may die of other causes^[1]^.

Endovascular aneurysm repair (EVAR) has become the primary treatment option in anatomically suitable patients with infrarenal aortic aneurysms. Randomised controlled trials have shown a reduced perioperative mortality rate compared with open repair^[2]^. The therapeutic success of EVAR is closely related to the evolution of renal function, which may compromise the results regarding the morbidity and mortality of the treatment. The monitoring of its evolution is fundamental to optimise the results and to detect possible complications or therapeutic failure^[3]^.

Several serum markers have been associated with perioperative risk. The relationship between C-reactive protein (CRP) and AAA has been studied, demonstrating a positive relationship between aneurysm-related CRP and the aneurysm’s size, and being a predictor of post-treatment aneurysm progression and a prognostic factor of treatment^[4]^.

Micro ribonucleic acids (miRNAs, also known as microRNAs or miR) were not known until the end of the 20^th^ century, but their discovery opened a large field of research. MiRNAs are important regulators of gene expression. Recent studies on their origin, biogenesis, and mechanisms of action have greatly increased the knowledge about their molecular biology^[5]^.

Due to their genetic regulation function in several biological processes and metabolic functions, as well as their high stability in biological fluids, circulating miRNAs have been highlighted as having great possibility for the identification of new non-invasive biomarkers of diseases^[6,7]^. In addition, they have been the target of many studies in order to understand their role in the pathophysiology of various diseases, including malignant neoplasms, atherosclerosis, and aneurysms^[8-10]^.

A serum analysis of miRNAs in patients with AAA identified 151 miRNAs with altered expression, with miRNA-1281 hyperexpressed (> 8-fold) in patients with aneurysm^[6]^. This motivate the inclusion of miRNA-1281 in the present study to analyze its evolution after EVAR and its role as AAA biomarker. This miRNA is evolved in many biological pathways including regulation of lipid metabolism by peroxisome proliferator-activated receptor alpha^[11]^.

The aim of this study was to evaluate the relationship between miRNA-1281 and CRP before and after the endovascular treatment of AAA, as well as the evolution of renal function, correlating with clinical data, aneurysmal sac change, and postoperative complications. Patients were evaluated before and six months after EVAR with clinical, laboratorial, and computed tomography (CT) examination. The planning for taking the samples for pre-treatment and six months later took into consideration the baseline of the disease to be treated and the furthest period from the procedure where there would be no further influence on the inflammatory process of the procedure and even any flow or morphological changes could have already appeared as, for example, the type II endoleaks.

## METHODS

We performed a prospective study of a single centre in the Division of Vascular and Endovascular Surgery of Clinical Hospital of Ribeirão Preto Medical School, University of São Paulo, Ribeirão Preto, São Paulo, Brazil during the period from January 2014 to November 2015. The institutional ethics committee approved the study and each patient provided signed informed consent prior to participation.

Inclusion criteria were as follows: patients with degenerative infrarenal AAA, with or without iliac aneurysms, eligible for standard endovascular repair were included in the present study, respecting current treatment indication^[12]^.

Exclusion criteria were as follows: patients with a ruptured aneurysm; patients with an aneurysm in any arterial territory other than the infrarenal aorta; patients who, for whatever reason, did not complete the total follow-up time of six months; patients with underlying diseases (medical psychosis, meningitis, stroke, multiple sclerosis, tumours, and autoimmune diseases); and patients who refused to participate in the study.

### Description of Devices

Six different types of endografts were used: AFX® (Endologix Inc, Irvine, California, United States of America [USA]) (n=9); GORE® EXCLUDER® (Excluder; W. L. Gore and Assoc, Flagstaff, Arizona, USA) (n=7); MEDTRONIC® ENDURANT® (Endurant; Medtronic, Minneapolis, USA) (n=7); HERCULES^®^ (MicroPort Scientific Corporation, Shanghai, China) (n=4); COOK® ZENITH® (Zenith; CookInc, Bloomington, Indiana, USA) (n=2); and ANACONDA^®^ (Anaconda; Sulzer Vascutek, Bad Soden, Germany) (n=1). The variety of endoprostheses used are justified by the variability of the anatomy of each aneurysm, with the most adequate endoprosthesis selected for each case.

### Laboratory Examinations

Peripheral venous blood samples were obtained 24 hours before the endoprosthesis implant and six months after the procedure. Whole blood was stored in a tube containing ethylenediaminetetraacetic acid (EDTA) and frozen at -80ºC. All of the collected material was stored until analysis.

### Ribonucleic Acid (RNA) Extraction

Total RNA was extracted with TRIzol^®^ (Thermo Fischer Scientific, Carlsbad, California, USA) reagent according to the manufacturer’s instructions. The blood samples were collected in EDTA tubes in quantities of approximately 4 mL. The samples were transferred to 50 mL falcon sterile tube free of ribonuclease, to which 20 mL of red cell lysis buffer was added. After maintaining the solution for 15 minutes on ice, it was centrifuged again for 10 minutes at 3000 rpm. The supernatant was discarded, leaving the pellet, which was resuspended in 250 mL of phosphate-buffered saline (or PBS) and 750 mL of TRIzol and transferred to a 1.5 mL Eppendorf tube. From this moment, the blood samples were processed according to the TRIzol protocol.

### MiRNA Complementary Deoxyribonucleic Acid (cDNA) Synthesis

Reverse transcription (RT) was performed using the commercial kit High Capacity cDNA Reverse Transcription (Applied Biosystems, Walthan, Massachusetts, USA). For the miRNA cDNA synthesis, for every 5 ng of RNA, we added 0.75 µl of RT Buffer, 0.075 µl of deoxynucleotide triphosphate, 1.5 µl of specific primers (miRNA or endogenous control), 0.5 µl of the MultiScribe^TM^ (Thermo Fischer Scientific, Carlsbad, California, USA) enzyme, and 0.094 µl of ribonuclease out (1.9U), making the solution up to a total volume of 7.5 µl with diethyl pyrocarbonate (DEPC)-treated water. The samples were then taken to a thermal cycler for 30 minutes at 16ºC, 30 minutes at 42ºC, five minutes at 85ºC, and cooled to 4ºC. Later, the samples were stored in a freezer at -20ºC. Before using the synthesised cDNA in the real-time polymerase chain reaction (PCR) reaction, it was diluted by 1:4 in DEPC-treated water.

### Real-time Quantitative Polymerase Chain Reaction (RQ-PCR) of MiRNAs

Real-time PCR was used to confirm the differential expression of miRNAs. TaqMan Assay-on-demand (Applied Biosystems, Walthan, Massachusetts, USA), which is commercially available, was used in the expression of quantitative analysis. The comparative CT method (∆∆CT) was used to calculate the relative quantification of expression. The U6 gene was used as a house-keeping gene. The amplification reactions by RQ-PCR were performed twice with the TaqMan Master Mix (Applied Biosystems, Walthan, Massachusetts, USA) reagent. The reaction had a final volume of 10 µl, including 5 µl of the specific TaqMan Master Mix reagent, 0.5 µl of each specific probe, and 4.5 µl of diluted cDNA. The 7500 Fast Real-Time PCR System (Applied Biosystems, Walthan, Massachusetts, USA) detection equipment was used with the 7500 Sequence Detection System (Applied Biosystems, Walthan, Massachusetts, USA) software in order to obtain the CT values. The standard amplification conditions were 95ºC for 10 minutes, followed by 40 cycles of 95ºC for 15 seconds and 60ºC for one minute. The data were then exported to Excel spreadsheets in order to calculate the values of 2-∆∆CT. A threshold of 0.1 was then established for the endogenous and studied miRNAs; the value considered for the deviation between samples was up to 0.5.

### Aneurysm Morphometry

The 32-bit Osirix^R^ program was used to perform aneurysm measurements, which were plotted in a previously prepared diagram. The measurements were performed at two time points (preoperatively and six months after the surgical procedure) by the same examinator previous the blood data analysis.

### Procedures

The procedures were performed with the patient under general anaesthesia by the same team for all patients and following the institutional protocols for vascular interventions. The procedures were performed in a surgical centre, using a GE/OEC 9900 ELITE (GE Hualun Medical Systems Co, Beijing, China) surgical arch, with the bilateral femoral access route for delivery and the release of devices. Non-fractioned heparin (5,000 units) was given intraoperatively shortly after the insertion of the introducer. For angiographic study, a low osmolarity non-ionic contrast agent (Iopamiron 300, Bracco Imaging SpA, Ferentino, Italy) was used.

### Follow-up

All patients were followed up at the same outpatient clinic, with returns scheduled for one, six, and 12 months (the patients had free access to outpatient clinics if necessary). At the return visits, they underwent a thorough clinical examination, performed by one of the team members, a trained vascular surgeon. These patients underwent surveillance (total aortic angiography by CT or magnetic resonance imaging) at three times: one, six, and 12 months after the surgical procedure, with the purpose of investigating leaks and/or changes in aneurysmal sac morphometry.

### Statistical Analysis

Statistical analysis was performed using GraphPad Prism version 6.0 (GraphPad Software Inc., San Diego, California, USA). Normal distribution was not assumed. Data are presented as median (interquartile range) unless otherwise stated. For the comparison of the demographic characteristics and clinical variables of the studied subgroups, the Fisher's exact test was used for the nominal variables, and the non-parametric Mann-Whitney test was used for the single continuous variable (age). Non-parametric Mann-Whitney and Wilcoxon tests were used for variables with non-normal distributions. In addition, the correlation was performed by stations, calculating the Spearman correlation coefficient (*r*). The null hypothesis was rejected when the chance of occurrence of the observed differences did not exceed 5% (*P*<0.05).

## RESULTS

Forty-one patients were recruited for the study, and 11 were excluded: surgical site infection (n=3 patients); accidental renal artery occlusion (n=1 patient); malignant neoplasm (n=1 patient); type II endoleak (n=3 patients); loss of follow-up (n=2 patients); death (n=1 patient). Thirty treated patients completed the study. Most of the patients were male elderly and smokers (Table 1). There was no need for transfusion in any of the cases. All procedures were well performed. Comorbidities are described in Table 2.

**Table 1:** Clinical and demographic data of included patients.

Variable	Minimum	Mean	Medium	Standard deviation	Maximum
Clearence	30.64	65.74	63.78	21.29	125.44
Creatinine	0.66	1.14	1.11	0.29	2.00
Aneurysm diameter	3.33	5.62	5.57	1.14	8.90
miRNA 1281	0.21	1.66	1.00	1.95	8.14
C-reactive protein	0.02	1.67	0.72	3.52	19.47
Total colesterol	60.00	179.90	179.00	51.99	287.00
Triglycerides	66.00	170.90	153.00	82.46	360.00
Age	50.00	68.60	68.00	9.52	83.00
Weight	45.00	73.80	70.00	17.03	118.00
Female	N	4.00			
	%	13.33			
Male	N	26.00			
	%	86.67			

miRNA=micro ribonucleic acid

**Table 2:** Comorbidities.

Comorbidity	N	%
Smoking	26	86.67%
SAH	20	66.67%
Dyslipidemia	8	26.67%
PAD	5	16.67%
Diabetes	5	16.67%

PAD=peripheral arterial disease; SAH=systemic arterial hypertension

**Fig. 1:**
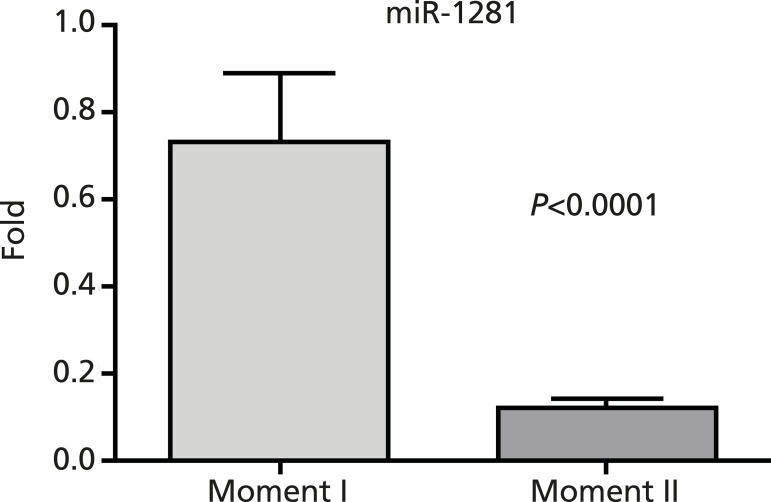
Micro ribonucleic acid (MiRNA)-1281 expression before and after endovascular aneurysm repair (EVAR). Moment I - before EVAR. Moment II - six months after EVAR.

The median increase of miRNA-1281 before the procedure was 1.66-fold, with values ranging from 0.21 to 8.14. For men, it was 1.64, and for women, it was 1.72. After endovascular treatment, it was 0.27 with *P*<0.0001 (Figure 1). Among males, it was 0.27, and among females, it was 0.30.

Creatinine baseline values > 1.5 mg/dL were considered as prior renal dysfunction. Overall, 16.6% of the patients presented an increase > 25% in creatinine serum level or drop in glomerular filtration > 20% after EVAR and were considered to have renal dysfunction (Table 2). The mean creatinine increased by 0.07 mg/dl (*P*=0.68).

Mean CRP levels before the procedure were 1.67 mg/L, with distant extreme values ranging from 0.02 to 19.47, and, after the procedure, 0.69 mg/L (*P*=0.48).

There was no statistically difference in total cholesterol or triglycerides levels during EVAR follow-up. Also, there was no relation between total cholesterol (mean 179±51, *P*=0.22) or triglycerides (170±82, *P*=0.8) and miRNA-1281 expression.

## DISCUSSION

When the UK Small Aneurysm Trial demonstrated no benefit from surgical treatment to small AAA, finding a clinical treatment to limit aneurysm growth became the target of several researches, especially in the last decade. However, there are no successful therapies in this field yet. Understanding the risk factors and pathological mechanisms of this disease is important to change this reality.

The existing literature on miRNA and AAA is still scarce, and the biological importance seems unclear. The prevalence of AAA is associated with advanced age, male gender, white race, positive family history, smoking, hypertension, hypercholesterolaemia, peripheral vascular occlusive disease, and coronary artery disease. The risk of developing an AAA increases considerably after the age of 60 years. AAA is present in 1% of men between the ages of 55 and 64 years and the prevalence increases from 2% to 4% every decade thereafter. In our study, we found a higher prevalence of AAA in men (86.67%), with a mean age of 68.6 years, in agreement with other studies that describe a prevalence of four to six times greater in men than in women^[13]^.

Smoking is the major risk factor for aneurysm formation and the over time of smoking seems to have a greater influence than smoking burden^[14]^. In our series, we found 26 smokers (86.67%), corroborating the literature data.

An important factor to consider in endovascular treatment is impairment of renal function. Some authors have reported no long-term effects on renal function, while others have found a significant decline over time^[15]^.

Renal function decline appears to be multifactorial and may be attributed to prior renal dysfunction, other comorbidities, the procedure itself, type of endoprosthesis, and diagnostic and therapeutic procedures required to evaluate and treat complications related to endoprosthesis during follow-up. In our series, creatinine levels and glomerular filtration rate showed no statistical variation before and after the procedure (*P*=0.68) from the preoperative period to a six-month follow-up.

The relationship between several serum markers and AAA has broadened the fields of the study, such as CRP and miRNAs. There is relationship between CRP and AAA, as a predictor of post-treatment aneurysm progression and as a treatment prognostic factor^[4]^. In our study, we also found significance in the positive association between CRP and aneurysm diameter (*P*=0.0009, *r*=-0.53044), which suggests that patients with bigger AAA present higher levels of CRP; these data might be related to a higher inflammatory activity in AAA with larger diameters.

Novel studies also demonstrated a significant increase in the plasma concentrations of fibrinogen, D-dimer, and the thrombin-antithrombin III complex in people with AAA, as well as a significant correlation between aortic diameter and D-dimer, in addition to these haemostasis markers. Other markers related to AAA include matrix metalloproteinase (MMP) 9, tissue inhibitor of MMP 1, interleukin 6, α1-antitrypsin, apolipoprotein A, and high-density lipoprotein. Despite a wide variety of biomarkers identified in patients with AAA, their prognostic value is yet to be established^[16]^.

In comparison to the usual protein biomarkers, miRNAs may provide early disease markers (usually the protein biomarkers are released after tissue damage) or those of greater specificity. In fact, miRNAs can be detected quantitatively by the RQ-PCR technique with high sensitivity, specificity, and multiplexing potential. However, RQ-PCR remains a relatively time-consuming technique compared to many analyses currently used in clinical laboratories, and nowadays there is no consensus on a method of data normalisation^[17]^.

Zhang et al.^[6]^ studied the expression of miRNAs in patients with AAA. They evaluated 10 cases with aneurysm and 10 controls through microarray in blood samples and concluded that of the 151 miRNAs that presented a difference of at least two-fold, three patients presented a large increase (> 5) (miRNA 191-3p, miRNA 455-3p, and miRNA 1281). In a second step, these results were confirmed in a larger cohort of patients (60 cases and 60 controls) through RQ-PCR, with a fold increase of 5.90, 5.44, and 3.09 for miRNA-191-3p, miRNA455 -3p, and miRNA-1281, respectively.

Maegdefessel et al.^[18,19]^ investigated the role of miRNA-29b in two murine models of experimental AAA: the porcine pancreatic elastase infusion model in C57BL/6 mice and the angiotensin II (AngII) infusion model in apolipoprotein E-deficient (*or ApoE^-/-^*) mice. AAA development was accompanied by decreased aortic expression of miRNA-29b, along with the increased expression of known miRNA-29b targets, *Col1a1, Col3a1, Col5a1*, and *Eln*, in both models. A similar pattern of reduced miRNA-29b expression and increased target gene expression was observed in human AAA tissue samples compared to that in organ donor controls, proposing that miRNA-29b can induce significant perivascular fibrosis of the aortic wall and thereby protect the aorta from expansion via transforming growth factor beta modulation. Using the same model, miRNA-21 increased as AAA develop, as nicotine enhances up-regulation of miRNA-21 and then phosphatase and tensin homologue activity.

More than biomarkers, miRNAs are promising targets in direct therapies to limit aneurysm growth. In a novel therapeutic approach, Wang et al.^[20]^ delivered a miRNA-126 mimic guided by microbubble ultrasound to an AAA AngII-induced mouse model to down-regulate vascular cell adhesion molecule 1 and prevent aneurysm development. In other research using the same murine model, the inhibition of miRNA-712 and human/murine homologue miRNA-205 as AngII-induced miRNAs in the abdominal aortic endothelium *in vivo* and *in vitro* significantly decreased the aortic MMP activity and inflammation, preventing AAA development^[21]^.

However, there are no studies evaluating such miRNAs as markers after treatment, which was the objective of our study. In our study, miRNA-1281 was identified in the whole blood of patients with AAA and showed a significant reduction in expression after endovascular treatment, with a significant decrease (> 5-fold) after the surgery (*P*<0.0001), showing a positive and significant correlation both with the presence of the aneurysm and with the favourable clinical and anatomical evolution. Additionally, there was no association of miRNA expression with the diameter of the aneurysm (*P*=0.7058). Our study suggests a limited restorative property of aneurysmal tissue with subsequent improvement following endovascular treatment because miRNA-1281 was overexpressed and significantly reduced its levels after endovascular treatment.

Recent studies have shown a relationship of some miRNAs with renal dysfunction, such as miRNA-148b, miRNA-25, and miRNA-30b, among others^[22]^. However, we did not find any studies that related miRNA-1281 to the regulation of renal function. In our study, creatinine levels before and after EVAR remained stable and presented no relation with miRNA-1281 expression.

Rotllan et al.^[23]^, in a recent study, demonstrated that the inhibition of miRNA-1281, miRNA-148a, or miRNA-185 led to a decline in low-density lipoprotein levels, but did not assess their relationship to total cholesterol and triglycerides^[23]^. In our study, we found no correlation between miRNA-1281 expression and total cholesterol or triglyceride levels.

Tarbase experimentally supported interactions for miRNA-1281 with a list of 67 genes. PubMed Central research shows 204 genes linked to AAA. The only common gene between both is related to the transcription factor activator protein 2 (AP-2) alpha pathway. AP-2α is a member of the AP-2 transcription factor family consisting of α, β, γ, δ, and ε subunits. Aspirin activates AMP-activated protein kinase (AMPK) to increase AP-2α in accelerated atherosclerotic plaques. This pathway is also related to AAA incidence and growth through AMPKα2/AP-2α signalling^[24,25]^. Therefore, miRNA-1281 may influence AAA growth and incidence through AP-2α, although more studies are needed to confirm this hypothesis. In our study, intervention resulted in a significant decrease in the expression of the miRNA-1281 after six postoperative months, suggesting that the exclusion of the aneurysmal sac changes the pathways of the miRNAs. However, our findings share the inexistence or weak correlation of the miRNAs with the diameter of the aneurysm. This finding was consistent with previous study using the same methodology but with different types of miRNAs.

The reason why miRNA-1281 reduces after aneurysm treatment is still ununderstood. Once the aortic wall disease was not removed in EVAR, we hypothesize that miRNA-1281 expression may be related to aortic wall stress or flow changes. However, this was not evaluated in this study and other experiments are needed to confirm that. A sudden reduction in hemodynamic stress in the aneurysmal wall could influence the expression of several molecules, including miRNA-1281, thus interfering with local lipoprotein metabolism correlating with aneurysmal sac stabilization after EVAR. This hypothesis would also require further studies for evaluation.

### Limitations

Several limitations of our study may be considered. The small number of patients, which is reflected in the large standard deviations; the use of previously described miRNAs not being performed a study of paired controls, which may not be reproducible since the miRNAs involved in the pathophysiology of AAA are not known; and the non-inclusion of a patient with endoleak, which impairs the real meaning of the reduced expression of miRNAs studied in the present study after endovascular treatment.

There is currently no available blood test for the diagnosis or monitoring of AAA. Given the stability of the miRNAs in circulation and their association with AAA, they are potential biomarkers for AAA. Further research is needed with rigorous case-control group definitions, sample acquisition protocols, and miRNA expression profiling techniques to identify miRNAs specific for AAA compared to other cardiovascular diseases for diagnostic, prognostic, and therapeutic purposes.

## CONCLUSION

The study shows a significant reduce of miRNA-1281 after six months of the EVAR treatment for AAA. These data indicate the need of new studies to verify miRNA-128 as a possible marker of EVAR follow-up, with a larger number of samples and including cases with endoleaks as well.

**Table:** 

Authors' roles & responsibilities	
LM	Substantial contributions to the conception or design of the work; or the acquisition, analysis, or interpretation of data for the work; drafting the work or revising it critically for important intellectual content; final approval of the version to be published
BR	Drafting the work or revising it critically for important intellectual content; final approval of the version to be published
EJRT	Drafting the work or revising it critically for important intellectual content; final approval of the version to be published
MSR	Drafting the work or revising it critically for important intellectual content; final approval of the version to be published
DT	Agreement to be accountable for all aspects of the work in ensuring that questions related to the accuracy or integrity of any part of the work are appropriately investigated and resolved; final approval of the version to be published
EEJ	Final approval of the version to be published
